# Supplementary material for: Parkinsonism Sac domain mutation in Synaptojanin-1 affects ciliary properties in iPSC-derived dopaminergic neurons
Source: bioRxiv. 2023 Oct 13:2023.10.12.562142. Preprint. [Version 2] doi: 10.1101/2023.10.12.562142 (PMC10592818; doi:10.1101/2023.10.12.562142)
Supplement: Supplement 1 [file NIHPP2023.10.12.562142v2-supplement-1.pdf]

bioRxiv preprint doi: https://doi.org/10.1101/2023.10.12.562142; this version posted October 13, 2023. The copyright holder for this preprint (which was not certified by peer review) is the author/funder, who has granted bioRxiv a license to display the preprint in perpetuity. It is made available under aCC-BY 4.0 International license.

Table S1. List of antibodies/dyes used in this study.

Target Protein	Company; Catalog number	Antibody species	Working dilution for immunofluorescence	Working dilution for immunoblotting
βIII-tubulin (TUJ1)	BioLegend; 801201	Mouse	1:500	1:3000
βIII-tubulin	Abcam; ab18207	Rabbit	1:500	N/A
α-Tubulin	Sigma Aldrich; T5168	Mouse	N/A	1:10000
Tyrosine Hydroxylase	EMD Millipore; 657012	Rabbit	1:300	N/A
Acetylated-tubulin	Sigma-Aldrich; T6793	Mouse	1:500	N/A
ARL13b	NeuroMab; N295B/66 75-287	Mouse	1:250	N/A
ARL13b	Proteintech;17711-1-AP	Rabbit	1:250	N/A
γ-tubulin	Sigma-Aldrich; T6557	Mouse	1:500	N/A
Adenylate Cyclase III	Abcam; 125093	Rabbit	1:200	N/A
Synaptojanin-1	Novus Biologicals; NBP1-87842	Rabbit	1:250	1:1000
Amphiphysin-2	EMD Millipore; 99D	Mouse	1:250	N/A
Clathrin light chain	EMD Millipore; AB9884	Rabbit	1:250	N/A
Auxilin	De Camilli Lab	Rabbit	1:250	N/A
Ub (FK1)	EMD Millipore; 04-262	Mouse	1:500	1:1000
Ub (FK2)	Enzo Life Sciences; BML-PW8810	Mouse	1:500	1:1000
$Ca_v1.3$	Alomone labs;ACC-005	Rabbit	1:250	N/A
DAPI	Thermo Fischer Scientific; D3571	N/A	1:1000 (stock: 1 mg/ml)	N/A
Synapsin I	Synaptic Systems; 106103	Rabbit	1:250	N/A
Synaptophysin	Synaptic Systems; 101002	Rabbit	1:500	N/A
Synaptophysin	Synaptic Systems; 101001	Mouse	1:250	N/A

bioRxiv preprint doi: https://doi.org/10.1101/2023.10.12.562142; this version posted October 13, 2023. The copyright holder for this preprint (which was not certified by peer review) is the author/funder, who has granted bioRxiv a license to display the preprint in perpetuity. It is made available under aCC-BY 4.0 International license.

Dopamine transporter	EMD Millipore; MAB369	Rat	1:500	1:1000
Vinculin	Sigma-Aldrich; V4505	Mouse	N/A	1:1000

bioRxiv preprint doi: https://doi.org/10.1101/2023.10.12.562142; this version posted October 13, 2023. The copyright holder for this preprint (which was not certified by peer review) is the author/funder, who has granted bioRxiv a license to display the preprint in perpetuity. It is made available under aCC-BY 4.0 International license.

Supplementary Figure 1

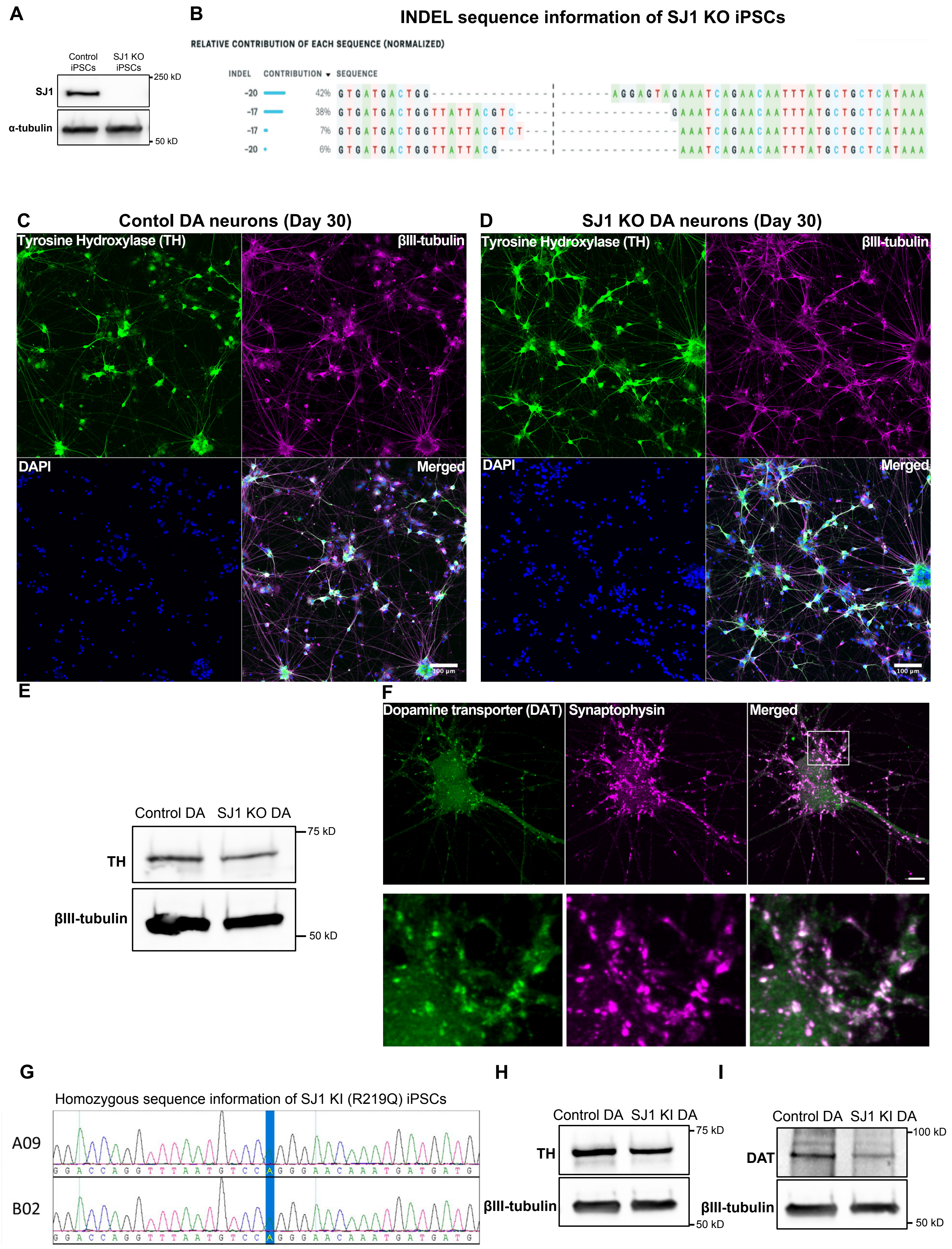

bioRxiv preprint doi: https://doi.org/10.1101/2023.10.12.562142; this version posted October 13, 2023. The copyright holder for this preprint (which was not certified by peer review) is the author/funder, who has granted bioRxiv a license to display the preprint in perpetuity. It is made available under aCC-BY 4.0 International license.

Supplementary Figure 1: Genetic and biochemical validation of SJ1 KO and SJ1^{RQ}KI iPSCs and iPSC-derived DA neurons

(A) Anti-SJ1 and anti-α-tubulin (loading control) western blot of control and edited WTC11 iPSCs. (B) Normalized relative contribution of INDEL sequences (normalized) of SJ1 KO iPSCs. (C and D) Fluorescence images of control and SJ1 KO iPSC-derived DA neurons (day 30) immunolabeled with antibodies directed against tyrosine hydroxylase (TH) (green) and βIII-tubulin (magenta). Nuclei were labeled with DAPI (blue)(Scale bars, 100 μm). (E) Anti-TH and anti-βIII-tubulin (loading control) western blot of control and SJ1 KO DA neurons (day 30). (F) Fluorescence images of control iPSC-derived DA neurons (day 30) immunolabeled with antibodies directed against dopamine transporter (DAT) (green) and synaptophysin (magenta). (G) Chromatogram sequences of two homozygous KOLF2.1 SJ1^{RQ}KI iPSC clones. (H) Anti-TH and anti-α-tubulin (loading control) western blot of control and edited KOLF2.1 iPSCs. (I) Anti-DAT and anti-βIII-tubulin (loading control) western blot of control and edited KOLF2.1 iPSCs.

bioRxiv preprint doi: https://doi.org/10.1101/2023.10.12.562142; this version posted October 13, 2023. The copyright holder for this preprint (which was not certified by peer review) is the author/funder, who has granted bioRxiv a license to display the preprint in perpetuity. It is made available under aCC-BY 4.0 International license.

Supplementary Figure 2

A Abnormal-looking cilia in SJ1 KO DA Neurons

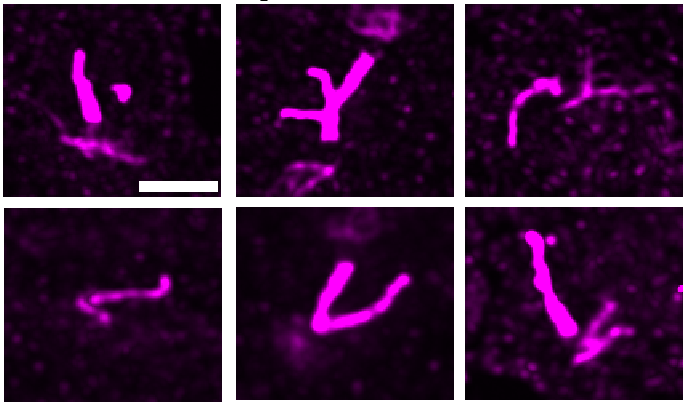

Supplementary Figure 2: SJ1 KO DA neurons show abnormal ciliary morphology

(A) Gallery of fluorescence images of Arl13b-labeled abnormal cilia from SJ1 KO iPSC-derived DA neurons. Scale bar, 2 µm.

Supplementary Figure 3

bioRxiv preprint doi: https://doi.org/10.1101/2023.10.12.562142; this version posted October 13, 2023. The copyright holder for this preprint (which was not certified by peer review) is the author/funder, who has granted bioRxiv a license to display the preprint in perpetuity. It is made available under aCC-BY 4.0 International license.

A SJ1 KI DA Neurons (#clone 2)

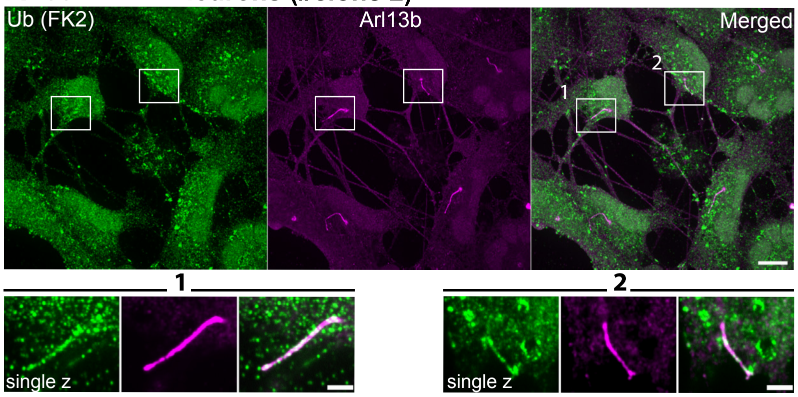

Supplementary Figure 3: Ubiquitination in SJ1^{RQ}KI DA neurons

(A) Fluorescence image of SJ1^{RQ}KI (B) DA neurons clone #2 immunolabeled with FK2 antibodies directed against lysine 63-linked ubiquitin chains (green) and against Arl13b (magenta). High magnifications of the boxed areas are shown below the main panels. (Scale bars, 10 µm; cropped areas: 2 µm).
